# Correcting the Error in Measuring Radiation Received by a Person: Introducing Cylindrical Radiometers

**DOI:** 10.3390/s19235085

**Published:** 2019-11-21

**Authors:** Robert D. Brown

**Affiliations:** Landscape Architecture and Urban Planning, Texas A&M University, College Station, TX 77843, USA; Robert.brown@tamu.edu; Tel.: +1-979-458-3192

**Keywords:** energy budget modeling, human thermal comfort, heat health, biometeorology, micrometeorology, microclimatology

## Abstract

Most human energy budget models consider a person to be approximately cylindrical in shape when estimating or measuring the amount of radiation that they receive in a given environment. Yet, the most commonly used instrument for measuring the amount of radiation received by a person is the globe thermometer. The spherical shape of this instrument was designed to be used indoors where radiation is received approximately equally from all directions. But in outdoor environments, radiation can be strongly directional, making the sphere an inappropriate shape. The international standard for measuring radiation received by a person, the Integral Radiation Measurement (IRM) method, yields a measure of the Mean Radiant Temperature (Tmrt). This method uses radiometers oriented in the four cardinal directions, plus up and down. However, this setup essentially estimates the amount of energy received by a square peg, not a cylinder. This paper identifies the errors introduced by both the sphere and the peg, and introduces a set of two new instrument that can be used to directly measure the amount of radiation received by a vertical cylinder in outdoor environments. The Cylindrical Pyranometer measures the amount of solar radiation received by a vertical cylinder, and the Cylindrical Pyrgeometer measures the amount of terrestrial radiation received. While the globe thermometer is still valid for use in indoor environments, these two new instruments should become the standard for measuring radiation received by people in outdoor environments.

## 1. Introduction

Global Climate Change (GCC) and Urban Heat Island Intensification (UHII) combined produce urban environments that are often hotter than the surrounding countryside. This can have negative effects on both chronic and acute health of urban residents. People are less likely to walk in overheated environments [1,2] and the US Surgeon General [3] has identified lack of walking as a key to chronic health problems. There is also an increase in morbidity and mortality during heat waves [4] and this increase can be linked to the physical characteristics of urban environments. Areas with higher amounts of tree canopies and less hard surfaces like asphalt and concrete have lower levels of acute health issues during heat waves [5]. In order to design urban environments that are safer during heat waves and more thermally comfortable at all times, urban planners and designers need valid and reliable evidence on which to base their designs. This has led to an increased interest in modeling and measuring the energy budgets of people in outdoor environments [6,7].

There are many models of outdoor thermal comfort and they have taken several different approaches. Johansson et al. [6] has called for a standardization in instruments and methods, while Coccolo et al. [7] conducted a comprehensive review and identified considerable variation in modeling approaches. Despite the different methods and approaches, there is almost universal agreement that, at the microclimate scale during hot weather, solar and terrestrial radiation have a substantial effect on the human energy budget. Models require accurate and precise estimates or measurements of radiation received; yet, the current instruments and models have built-in error.

Johansson et al. [6] identified several methods for determining the amount of radiation received by a person in an outdoor environment. This value is often reported as the mean radiant temperature (Tmrt), which is defined as the “uniform temperature of a surrounding surface giving off blackbody radiation, resulting in the same energy gain of a human body, given the prevailing radiation fluxes” [8] (p. 323) and is essentially the sum of all the solar and terrestrial radiation received by a person in a given environment. Brown and Gillespie [9] used a more easily-understood value of radiation absorbed by a person (Rabs), which is essentially the inverse of Tmrt.

**Figure 1:**
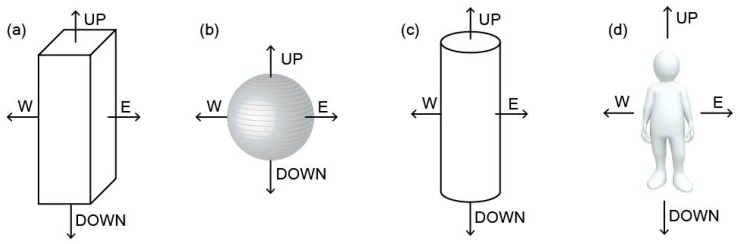
The shape of the three instruments/methods for measuring radiation received by a person in a landscape. (**a**) The Integral Radiation Measurement (IRM) as described by Thorsson et al. [10] measures radiation in each of the four cardinal directions, plus upward and downward. This essentially describes an extruded ‘peg’. (**b**) The globe thermometer is spherical and absorbs radiation equally from all directions. A thermometer inside the globe provides an average temperature that is translated into a Mean Radiant Temperature (Tmrt) value through an equation. (**c**) The cylindrical radiation thermometer [9] measures the amount of radiation absorbed by a vertical cylinder but relies on an empirical equation to estimate the amount of convective cooling. (**d**) The shape of a typical person is more similar to a cylinder than to a peg or a sphere.

Thorsson et al. [10] reported that Integral Radiation Measurement (IRM) is the most accurate method for determining Tmrt, but identified instrumental overestimates at high solar incidence angles and underestimates at low angles. This method involves measuring solar and terrestrial radiation simultaneously in six directions with directional weightings of 0.22 for each of north, south, east, west, and 0.06 for each of up and down directions [11]. This essentially measures the radiation received by an extruded square “peg” (Figure 1).

Johansson et al. [6] identified the globe thermometer as another method of estimating Tmrt but said that it tends to overestimate Tmrt during shady conditions and underestimates it in sunny weather. This instrument is spherical in shape, which does not look very much like a typical human body (Figure 1).

Humans arguably are neither square pegs nor spherical globes, but are more typically cylindrical in shape (Figure 1) and they have been commonly modeled as such [9,12].

These three different shapes of instruments in the same environment will yield different values of solar and terrestrial radiation received. The Cylindrical Radiation Thermometer (CRT) has a shape that most closely resembles a typical human, but it relies on an empirical heat flux equation [9] that can lead to errors in low or high wind situations.

The goal of this paper was therefore twofold: (1) To compare, through the use of three-dimensional geometrical analysis, the radiation received by square pegs, spheres, and cylinders in outdoor environments; and (2) to identify a more accurate and precise method of measuring the radiation received by people in outdoor environments.

## 2. Materials and Methods

The three instrument shapes (square peg, globe, and cylinder) were assessed in terms of their geometry and how their shape influences the measurement of solar and terrestrial radiation. For these tests, the up and down directions will not be included. Similarly, diffused and reflected radiation will not be included. The goal was not to provide a complete assessment of the radiant environment, but rather to illustrate situations that cause errors. Each instrument was placed first in a theoretical horizontal flat plane with no objects to obstruct or reflect the solar radiation, and secondly with a sun-warmed wall to (i) the north of the instrument; and (ii) to the north-east of the instrument. The wall was heated by incident solar radiation during the day and emitted terrestrial radiation. The amount of solar and terrestrial radiation received by each shape at test times of the day and with the wall in test locations are calculated and the results from the different shaped instruments are compared.

## 3. Results

### 3.1. Geometric Assessment of Solar Beam Radiation at Different Solar Angles

#### 3.1.1. Square Peg (IRM)

**Figure 2:**
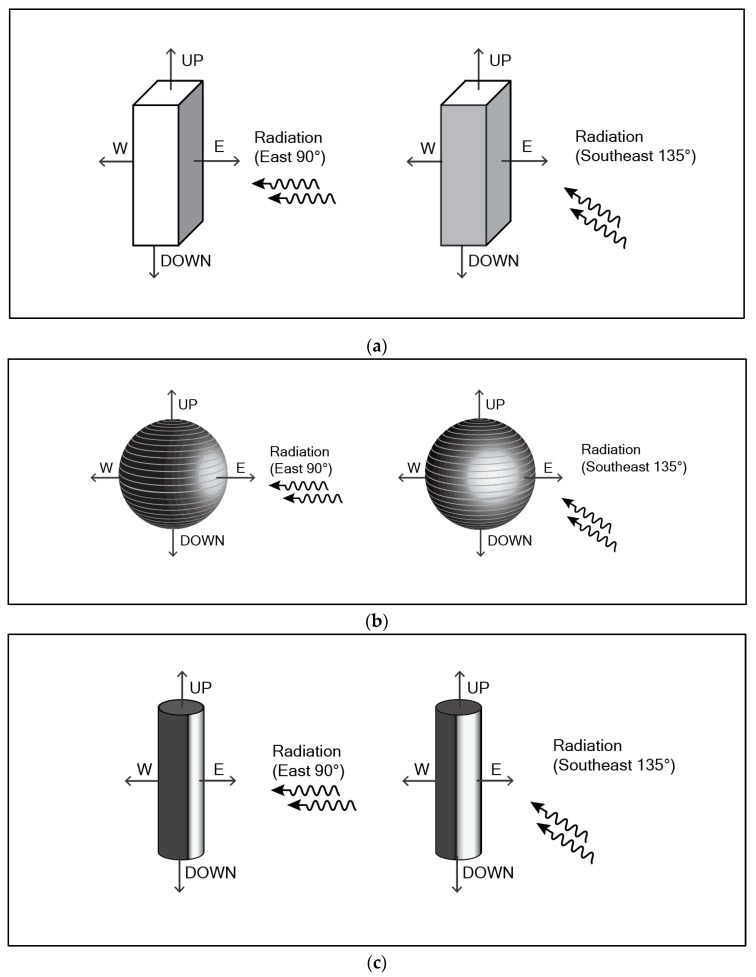
A theoretical environment where each of the instruments is located on a horizontal flat plane with nothing to obstruct the solar radiation beam. (**a**) The IRM method yields different values depending on the orientation of the instrument. (**b**) The globe thermometer yields overestimate at high sun angles and underestimates at low sun angles. (**c**) The cylindrical radiation thermometer provides a result that is the most similar to what a person would receive.

Low Sun Angle: When the sun rises exactly in the east and provides 400 Wm^−2^ of beam radiation on a vertical flat plate, the IRM pyranometer facing directly toward the east will record 400 Wm^−2^, while the other three instruments—facing south, west, and north—will all record 0 Wm^−2^ beam radiation. The average amount of beam radiation received by the four vertical faces is 100 Wm^−2^ (Figure 2a).

If the sun rises instead in the southeast with the same 400 Wm^−2^ beam radiation, the beam will hit both the east-facing and south-facing radiometers at 45°, so each one will measure 400 × cos45° = 283 Wm^−2^. The other two radiometers measure 0 Wm^−2^ beam, so the average of the four radiometers is 141 Wm^−2^ beam (Figure 2a).

The orientation of the instruments resulted in a 41% difference in measurement from the same amount of solar radiation (Table 1).

High Sun Angle: When the sun is directly south of the instruments at solar noon, the south-facing instrument will receive radiation according to Lambert’s Cosine Law [13]:

Φ = Φ_0_ cos Θ (1)

where:

Φ = flux density at the surface (Wm^−2^)
Φ_0_ = flux density normal to the beam (Wm^−2^)
Θ = angle between beam and normal to the surface

In the case of a very clear day when the sun is providing 1000 Wm^−2^ of beam radiation with an elevation angle of 45° above the horizon, the south-facing instrument will receive 707 Wm^−2^ of directional radiation, while the other three radiometers will receive 0. The average amount received will be 177 Wm^−2^.

If this same 1000 Wm^−2^ was received when the sun was 15° east of south, then the east-facing instrument will receive 707 cos(90−15) = 183 Wm^−2^ while the south-facing instrument will receive 707 cos(90−75) = 682 Wm^−2^. The west- and north-facing instruments will receive 0. The average amount of beam radiation measured will be 216 Wm^−2^. This is a 22% difference from the same amount of radiation received directly from the south (Table 1).

#### 3.1.2. Spherical Globe Thermometer

Low Sun Angle: Whether the sun rises in the east or southeast will have no effect on the beam radiation received by the spherical globe thermometer. The 400 Wm^−2^ is distributed over the surface of the sphere. The surface area of the sphere is four times the cross-sectional area, so the instrument will measure 100 Wm^−2^ (Figure 2b).

High Sun Angle: Similarly, when the sun is high in the sky to the south, the ratio will remain the same. The amount of solar radiation received by a circular flat plate is divided by 4 to yield the average amount received by the sphere. Thus, when the sun provides beam radiation of 1000 Wm^−2^, the average receipt of the sphere is 250 Wm^−2^.

#### 3.1.3. Vertical Cylinder

Low Sun Angle: When the sun rises in the east and provides 400 Wm^−2^ of beam radiation on a vertical flat plate, the direct beam hitting a cylinder is absorbed according to the cross-sectional area, which is simply diameter × length. It will be spread over the surface area of the cylinder, which is diameter × length × pi, so the capture area = 1/pi = 0.32. Thus, it will receive 400 × 0.32 = 128 Wm^−2^ no matter where the sun rises (Figure 2c).

High Sun Angle: Whether the sun is in the south or 15° east of south, the cylinder will receive the 1000 Wm^−2^ of radiation according to the cosine law, so it will receive 707 Wm^−2^. This amount will be spread over the surface area of the cylinder, so it receives 707 × 0.32 = 226 Wm^−2^ whether the sun is in the south or 15° east of south.

### 3.2. Geometric Assessment of Terrestrial Radiation

This assessment considers two situations: (a) A wall to the north of the instruments, and (b) a wall to the north-east of the instruments. The two walls are considered to be the same temperature as heated up by absorbed solar radiation and are the same distance from the instruments. The location of the wall will have a similar impact on the amount of terrestrial radiation received by a person in that location in a landscape and the orientation of the instrument or the wall does not influence the result.

For this test example, the ground is considered to be covered with grass and is at 25 °C; the wall fills 50% of the sky hemisphere and is 50 °C, while clear sky fills the other 50% of the sky hemisphere. For illustration purposes, the emissivity of surfaces is assumed to be 1.0 for all cases.

#### 3.2.1. Square Peg (IRM)

If the wall to the north of the instrument has a surface temperature of 50°C, according to the Stefan-Boltzmann equation, it will be emitting (273.15 + 50)^4^ × 5.67 × 10^−8^ = 618 Wm^−2^. The ground at 25 °C will be emitting 448 Wm^−2^ and the radiation from the sky can be estimated from Equation [12]:

Lsky = 213 + 5.5 Ta(2)

**Figure 3:**
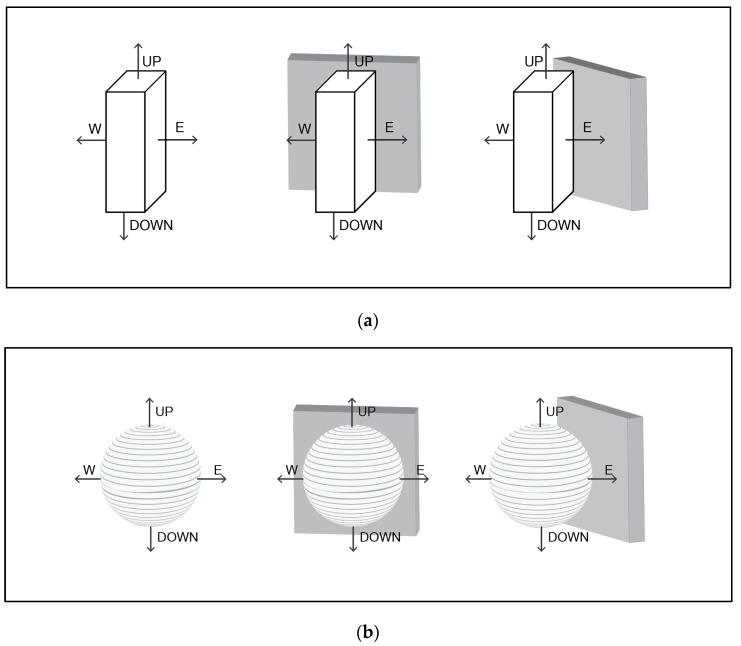

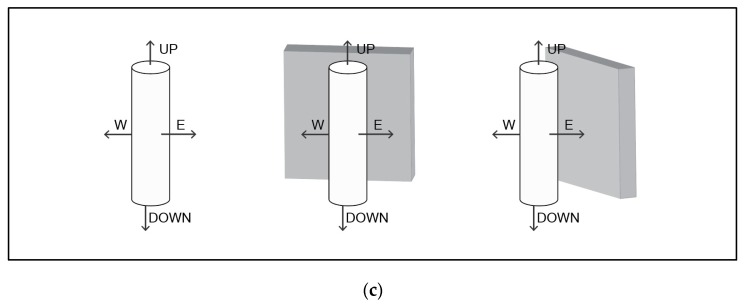
A theoretical environment where each of the instruments is set in an environment with a vertical wall to the north and the northeast. The vertical wall emits terrestrial radiation that is measured by each instrument. (**a**) The IRM underestimates the value in both cases; (**b**) the globe thermometer yields the correct value in all cases (which is why it is appropriate for use in indoor environments); and (**c**) the cylindrical radiation thermometer is not dependent on the orientation of the instrument and yields the same value at the globe thermometer.

In this case, the sky will emit 350.5 Wm^−2^. The north-facing pyrgeometer will register 618 Wm^−2^ while the south, east, and west facing pyrgeometers will record 0.5 × 448 + 0.5 × 350.5 = 399 Wm^−2^. Averaging the four values yields 454.75 Wm^−2^ (Figure 3a).

When the wall is to the northeast of the instrument, the north- and east-facing pyrgeometers will receive 50% from the ground, 25% from the sky, and 25% from the hot wall or 5 × 448 + 25 × 350.5 + 25 × 618 = 466.1. The south- and west-facing pyrgeometers will each receive 399 Wm^−2^. The average value will be 432.6 Wm^−2^.

#### 3.2.2. Spherical Globe Thermometer

The bottom half of the globe will receive directional terrestrial radiation from the ground = 448 Wm^−2^. The upper half of the globe will receive 50% from the wall and 50% from the sky = (0.5 × 618) + (0.5 × 350.5) = 484.25 Wm^−2^. The average of these two values is 466.1 Wm^−2^. The location of the wall relative to the globe does not change this value (Figure 3b).

#### 3.2.3. Vertical Cylinder

Considering the sphere of influence on the cylinder, half of the terrestrial radiation will be received from the ground and half from the sky hemisphere. The ground portion will be 448 Wm^−2^. The sky portion will be half from the sky and half from the wall = (0.5 × 350) + (0.5 × 618) = 484.25. The average will be 466.1 Wm^−2^ and as with the globe, the location of the wall relative to the cylinder does not change the amount of terrestrial radiation received (Figure 3c).

The different amounts of terrestrial radiation yielded by the three different shaped instruments is shown in Table 2.

## 4. Discussion

As reported in the literature [6,10] measurements taken by the IRM and the globe thermometer introduce small but important errors into the estimates of the amount of radiation a person receives in an outdoor environment. The geometric basis for these errors has been identified in this study. The cylindrical radiation thermometer, because of its shape, has the most appropriate geometry to represent a person, but it relies on an empirical convective heat loss equation to estimate the radiation received. What is needed is an instrument in the shape of the cylinder that does not require an empirical equation. This paper proposes new instrument designs that will correct the error and offer accurate and precise values of radiation received by a person in a landscape.

## 5. Proposal for New Instruments to Directly Measure Solar and Terrestrial Radiation of a Cylinder

This study proposes two new radiometers based on the same principles as the standard pyranometer and pyrgeometer; however, rather than measuring the radiation on a horizontal flat plate, they measure the amount of radiation received by a cylinder.

**Figure 4:**
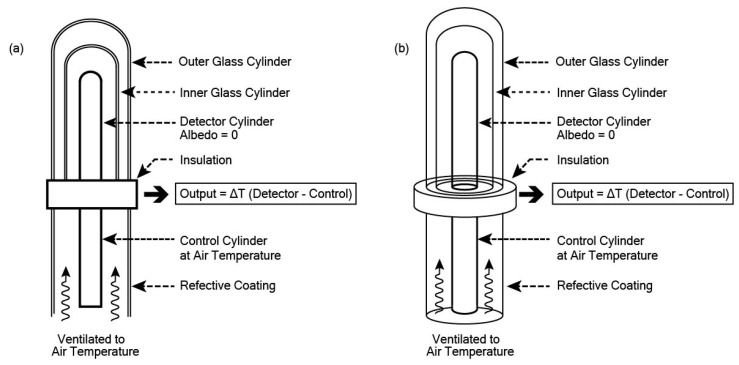
Design of a Cylindrical Pyranometer. (**a**) Cross-sectional view illustrates the relationship between the detector and the double glass shield; (**b**) Perspective view shows the cylindrical form of all components of the pyranometer.

The Cylindrical Pyranometer (Figure 4) consists of a black cylinder with very low albedo encased in a slightly larger double glass cylinder. This allows solar radiation to pass through but will negate other effects, such as convective heat flux and terrestrial radiative flux. There is an array of fine wire thermocouples embedded just below the black skin mid-way up the cylinder and wired in series. The signal provides an average temperature of the cylinder, which, when compared with the control temperature, can be translated into incoming solar radiation in Wm^−2^.

**Figure 5:**
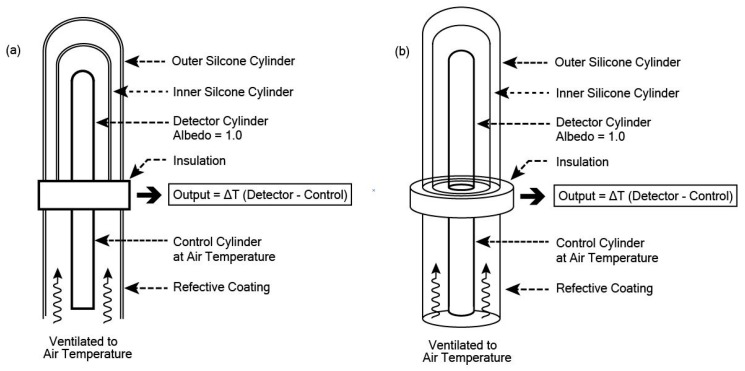
Design of a Cylindrical Pyrgeometer. (**a**) Cross-sectional view illustrates the relationship between the detector and the double silicon shield; (**b**) Perspective view shows the cylindrical form of all components of the pyrgeometer.

The Cylindrical Pyrgeometer (Figure 5) consists of a black cylinder with very low albedo and very low emissivity, but instead of glass, the enclosing cylinder is made of silicone with a thin film coating on the insider to block solar radiation. The signal provides an average temperature of the cylinder, which can readily be translated into Wm^−2^.

Instruments such as these radiometers will have a built-in uncertainty. It can be expected that these two instruments will have uncertainties similar to that of high-quality flat plate pyranometers and pyrgeometers, which are in the range of 3% to 5%.

## 6. Conclusions

Instruments that are currently being used to measure and estimate radiation received by a person in outdoor environments yield incorrect results. Two new radiometers, a cylindrical pyranometer and a cylindrical pyrgeometer, will provide more accurate and precise measurements of the radiation a person receives in a landscape.

With more than 80% of the American population and more than 50% of people worldwide living in cities, it is critical that urban environments be designed to provide thermally comfortable environments for people. Radiation is a critical component of the energy budget of people in urban outdoor areas, particularly during extreme heat events, and it is important that design and planning decisions are made based on accurate and precise measurements.

## 7. Patents

The two instruments described in this study have been registered and are “patent pending”.

## Figures and Tables

**Table 1:** Direct beam solar radiation received by each of the instruments/methods at low and moderate sun angles. The Integral Radiation Measurement (IRM) value is dependent on the orientation of the instrument, while the globe thermometer yields values lower than the cylinder at low sun angles and higher than the cylinder at higher sun angles.

Solar Elevation	Solar Azimuth	Beam (Wm^−2^)	IRM (Wm^−2^)	Globe (Wm^−2^)	Cylinder (Wm^−2^)
0°	East (90°)	400	100	100	128
0°	Southeast (135°)	400	141	100	128
45°	15° East of South (165°)	1000	216	250	226
45°	South (180°)	1000	177	250	226

**Table 2:** Terrestrial radiation received by each of the instruments when set beside a hot wall to the north and to the northeast. The IRM result is dependent on the orientation of the instrument, while the globe and cylindrical thermometers yield the same value.

Location of Wall	IRM (Wm^−2^)	Globe (Wm^−2^)	Cylinder (Wm^−2^)
North (0°)	454.75	466.1	466.1
Northeast (45°)	432.6	466.1	466.1
